# Polymer-Derived Boron Nitride: A Review on the Chemistry, Shaping and Ceramic Conversion of Borazine Derivatives

**DOI:** 10.3390/ma7117436

**Published:** 2014-11-21

**Authors:** Samuel Bernard, Philippe Miele

**Affiliations:** Institut Europeen des Membranes (IEM), UMR 5635 (CNRS-ENSCM-UM2), Universite Montpellier 2, Place E. Bataillon, Montpellier F-34095, France; E-Mail: philippe.miele@univ-montp2.fr

**Keywords:** boron nitride, borazine, composites, B-tri(methylamino)borazine, polyborazylene, poly[B-tri(methylamino)borazine], shaping, pyrolysis

## Abstract

Boron nitride (BN) is a III-V compound which is the focus of important research since its discovery in the early 19th century. BN is electronic to carbon and thus, in the same way that carbon exists as graphite, BN exists in the hexagonal phase. The latter offers an unusual combination of properties that cannot be found in any other ceramics. However, these properties closely depend on the synthesis processes. This review states the recent developments in the preparation of BN through the chemistry, shaping and ceramic conversion of borazine derivatives. This concept denoted as Polymer-Derived Ceramics (PDCs) route allows tailoring the chemistry of precursors to elaborate complex BN shapes which cannot be obtained by conventional process. The effect of the chemistry of the molecular precursors, *i.e.*, borazine and trichloroborazine, and their polymeric derivatives *i.e.*, polyborazylene and poly[tri(methylamino)borazine], in which the specific functional groups and structural motifs determine the shaping potential by conventional liquid-phase process and plastic-forming techniques is discussed. Nanotubes, nano-fibers, coatings, monoliths and fiber-reinforced matrix composites are especially described. This leads to materials which are of significant engineering interest.

## 1. Introduction

In the category of III-V nitrides, boron nitride (BN) represents an important compound which is currently considered as a wide gap semiconductor with a band gap energy corresponding to the UV region. BN is isoelectronic to carbon and thus, in the same way that carbon exists as diamond and graphite, boron nitride can be synthesized in the tetrahedrally structure (cubic BN, c-BN) and in a layered structure (hexagonal; h-BN). In the cubic crystalline form, alternately linked boron (B) and nitrogen (N) atoms form a tetrahedral bond network, exactly like carbon atoms do in diamond. In h-BN, the in-plane B and N atoms are bounded by strong covalent bonds while the out-of-plane layers are held together by Van der Waals forces. In comparison to graphite, the layered BN hexagons are arranged vertically and each nitrogen atom is surrounded by two boron atoms of the adjacent layers [1]. Hexagonal BN (h-BN but expressed in the following discussions as BN) is the most widely studied polymorph. It is the focus of the present paper.

BN is a synthetic binary compound discovered in the early 19th century and developed as a commercial material at the latter half of the 20th century [1,2]. This form shows similar physical properties analogous to graphite and like graphite the different bonding cause high anisotropy of the properties of BN. It displays good lubricating properties according to its layered structure (weakly held layers can slide over each other). It displays a very high thermal conductivity (in the direction of hexagons), a high thermal stability, a high resistance to corrosion and oxidation as well as a strong UV emission [3,4,5,6,7,8]. Depending on its shape and synthesis procedure, it can offer superhydrophobicity [6,9]. It is not wetted by most molten metals such as aluminium, iron and copper as well as hot silicium, glasses and salts and hence has a high resistance to chemical attack. It is nontoxic and it has good environmental compatibility. Unlike graphite, it offer high dielectric breakdown strength and high volume resistivity. Furthermore, the local polar character of the B-N bonds is present in the BN structure. Very recently, BN has been demonstrated to exhibit enhanced sorption properties of various substances such as organic pollutants [7,9,10,11,12,13,14,15,16] and hydrogen [16,17,18] due to the dipolar fields near its surface. However, these properties vary drastically with its crystalline quality (amorphous → turbostratic → hexagonal) which in fact is governed by the synthesis route employed to prepare BN.

The fabrication of BN is usually made by metallurgical-type pyrolysis reactions, using boric acid/oxide as a source of B and urea or melamine as a source of N with a catalyst such as calcium carbonate to promote crystallization [1,19]. Such conventional processes do not provide the possibility to produce relatively complex shapes and morphologies and usually involve the presence of impurities (because of the use of sintering additives to consolidate the materials) which affect the properties. One of the ways to improve ceramic materials in terms of compositional, structural homogeneities and to create a large variety of shapes is to control the structure of ceramics at very small length scales in an early stage of the fabrication. Chemistry may be the way to achieve this goal.

**Figure 1:**
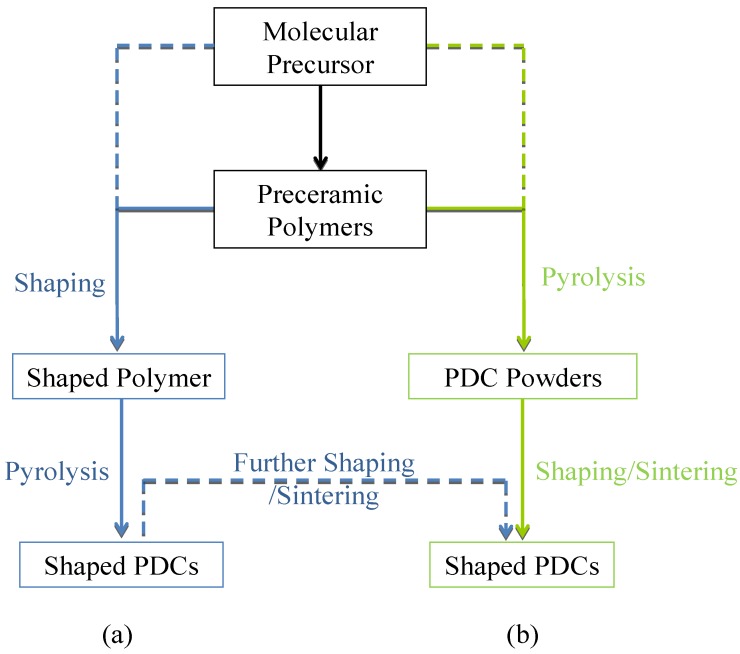
Overall process for preparing Polymer-Derived Ceramics (PDCs): (**a**) polymer shaping and pyrolysis; (**b**) polymer pyrolysis and PDC powders shaping/sintering.

Highly pure synthetic precursors in which uniform chemical composition is established at molecular scale are making an increasingly important contribution to the research development and manufacture of ceramic materials. This concept has two major advantages compared to conventional ceramic powders processing using solid-state reactions between powder reactants. The first advantage is to generate materials with enhanced physical and chemical properties and possibly new properties. The second advantage is to develop ceramic objects, for example coatings, monoliths, fibers, with tailored textural and structural properties. This method is called the Polymer-Derived Ceramics (PDCs) route. It is illustrated in Figure 1.

The PDCs route is an attractive means for the design of advanced ceramics with a compositional and structural homogeneity; in particular in non-oxide systems [20,21,22,23,24]. The main motivation to implement the PDCs route lies in the special advantages that are offered by molecular or polymeric precursors in controlling ceramic compositions and micro-/nanostructures (amorphous, crystalline or nanocomposite) and in processing materials in particular shapes and morphologies (dense or porous) that are difficult, or even impossible to obtain by conventional routes. As an illustration, this synthesis method has been applied to the preparation of simple carbides, nitrides of various main groups and transition elements [25,26,27] as well as homogeneous mixtures or solid solutions of pseudo-binary combinations of ceramics and nanocomposites [28,29,30,31]. It represents a synthetic approach in which the chemistry of molecular precursors is designed at atomic scale to deliver the desired inorganic polymer (called preceramic polymer) composition. Using a first strategy (Figure 1a), which is the most common, polymers synthesized from molecular precursors (chemically or thermally) may be shaped to produce after crosslinking and pyrolysis a large variety of compositions in particular shapes including porous materials [32,33,34], as well as nano-fibers [35,36,37], dense/membrane coatings [38,39], bulk parts [40,41] and composites [42], MEMS [43] and microfluidic systems [44] for a large range of application fields. It should be mentioned that polymerization and shaping can be achieved in parallel directly from molecular precursors. Through a second strategy (Figure 1b), the polymers (or molecular precursors) may be directly pyrolyzed into PDC powders. Then, the PDCs route can be coupled with more conventional shaping processes such as pressure-assisted sintering techniques to produce shaped PDCs such as monoliths [45,46,47]. Furthermore, if these pressure-assisted sintering techniques are coupled with the strategy 1 (Figure 1a), monolithic porous materials can be further produced [48,49].

In the categories of PDCs, a wealth of studies has been published on polymer-derived BN [1,50,51,52,53,54]. Here, we review the use of BN precursors suitable for various shaping process such as conventional liquid-phase process and plastic-forming techniques, especially those prepared from borazine derivatives. It is of high scientific and technological interest, because of the wide range of applications potentially offered by these materials in energy and environmental science. We felt that it was desirable and timely to present a comprehensive, detailed and critical review of the subject covering recent developments up to mid-2014.

The main reports dedicated to precursor-derived BN materials focuses on the use of borazine and trichloroborazine as BN precursors. These precursors offer reactive sites, *i.e.*, BH, NH, BCl bonds, to synthesize preceramic polymers, *i.e.*, polyborazylene and poly[tri(methylamino)borazine] in particular, which can be applied in the strategies depicted in Figure 1.

## 2. Borazine and Polyborazylene-Derived BN

**Figure 2:**
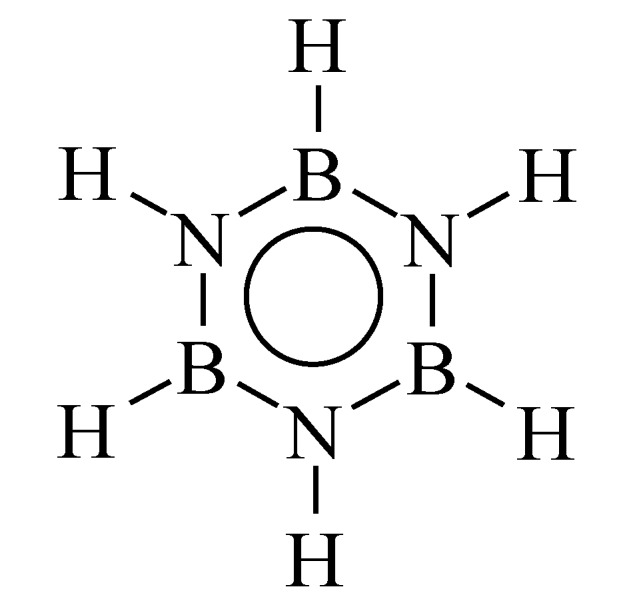
Borazine structure.

The selection of the BN precursor is important and precursors with the good B:N ratio while hydrogen (H) is the only element added to B and N are preferred to prepare BN. According to the presence of hydrogen linked to B and N, the correct B-to-N ratio and a preformed B–N-like ring structure (planar six numbered hexagonal ring), borazine H_3_B_3_N_3_H_3_ appears to be the ideal molecular precursor of BN (Figure 2).

Borazine (Figure 2) was originally discovered by Alfred Stock in 1926 [55]. It is economically competitive and attractive from a technical point of view, based on its reaction starting from cheap compounds, such as (NH_4_)_2_SO_4_ and (NaBH_4_), reacting in tetraglyme at low temperature (120–140 °C) [56]. Furthermore, only light gases such as dihydrogen and ammonia are expected to be evolved during the borazine-to-BN transformation. BN is in general obtained at relatively low temperature (1450 °C) without the necessity to use specific treatments such as ammonia treatment (use with oxygen and/or carbon-containing precursors to introduce nitrogen in the materials while oxygen and/or carbon are removed) and chemical etching when alkali metal-based precursors are used. However, borazine is highly volatile. Therefore, borazine is firstly self-condensed at low temperature inside an autoclave to generate a preceramic polymer, *i.e.*, polyborazylene, with a controlled molecular weight, physical state and composition according to the parameters fixed during the polymerization following the common PDCs strategy (*Polymerization* → *Shaping* → *Crosslinking* → *Pyrolysis*, Figure 1a).

**Figure 3:**
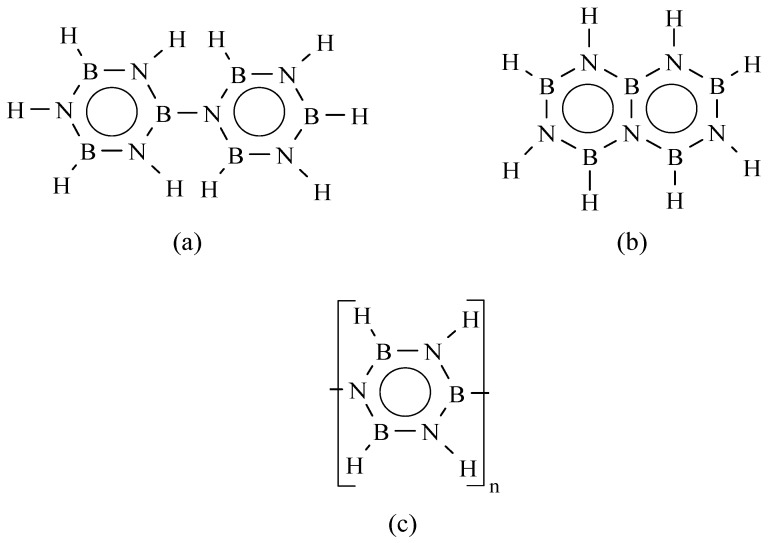
(**a**) Biphenyl and (**b**) naphtalenic-type units generated during the self-condensation of borazine leading to (**c**) polyborazylene structure.

A large number of studies has been focused on the self-condensation of borazine and the identification of polymerization mechanisms [57,58,59,60,61,62,63,64,65,66,67,68,69]. In the fifties and sixties, several authors studied the self-condensation of borazine. It generates biphenylic and naphtalenic-type structures (Figure 3a,b) through condensation reactions of BH and NH units as well as probable ring-opening mechanisms in the polyborazylene (Figure 3c) [60,61,62,63].

Sneddon *et al.* [64,65,66] were the first authors to isolate a solid polyborazylene through the self-condensation of borazine under vacuum at 70 °C for approximately 48 h. Elemental analyses of the compound suggested the formation of a branched-chain or partially cross-linked structure. Despite this crosslinked structure, this polyborazylene was soluble and appeared an excellent precursor to prepare fiber coatings [66]. Polyborazylene delivered BN by pyrolysis under argon or ammonia in the temperature range 900–1450 °C in excellent chemical (89%–99%) and ceramic yield (84%–93%). The increase of the temperature increased the density of BN as well as the crystallinity of samples based on X-ray diffraction investigations. However, the structure of BN remained turbostratic. This did not affect the stability in air of polyborazylene-derived BN. In air, polyborazylene-derived BN is stable up to 900 °C (no weight changes were observed by TGA in air). Above 900 °C, weight gain occurred to form boron oxide. The excellent stability in air of polyborazylene-derived BN was confirmed by Economy *et al.* [67].

**Figure 4:**
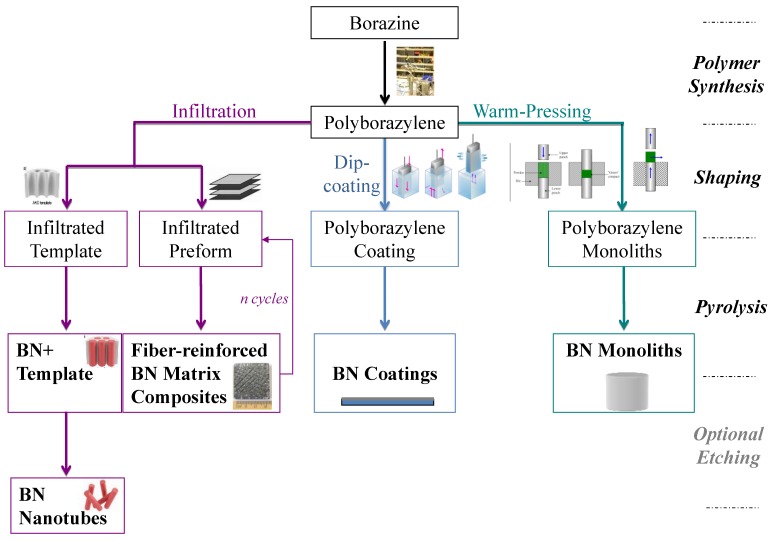
Examples of shaped BN derived from polyborazylenes.

Economy *et al.* [67] reported in 1993 the preparation of C/BN composites from a viscous polyborazylene which was obtained by self-condensation of borazine in an autoclave in a nitrogen atmosphere at 70 °C for 40 h. The as-obtained viscous polymer displayed a chemical formula of B_3.0_N_3.6_H_3.7_. In another paper [68], authors prepared the first inorganic mesophase by low-temperature thermolysis of the borazine. Indeed, based on the synthesis procedure reported by Sneddon *et al.* [66] as well as on the prediction made on the formation of biphenyl and naphtalenic-type units and requirements for the formation of pitch mesophases, authors modified the thermolysis rate to maintain certain mobility in as-formed molecules, and therefore obtain optically anisotropic phases during thermolysis of borazine. The formation of a liquid-crystalline phase during thermolysis provided an efficient way to produce a final BN material with a high degree of crystalline order on heating to 1800 °C. In particular, TEM of BN revealed the presence of a polycrystalline microstructure with a long-range preferred orientation [68]. In 2001, Babonneau *et al.* [69] reported the structural characterization of polyborazylene by ^15^N and ^11^B solid-state NMR. They identified the presence of two types of boron sites including *B*HN_2_ and *B*N_3_ and two types of nitrogen sites (*N*HB_2_ and *N*B_3_) as well as eight-members rings in addition to the expected six-members borazine rings in a solid polyborazylene. Through the study of the self-condensation of borazine at low temperature (45–60 °C) under argon in an autoclave, our group demonstrated the possibility to control the physical state of polyborazylene from liquid (45–55 °C) state (with viscosity increasing with the increase of the synthesis temperature) to solid state (≥60 °C) by adjusting the temperature of thermolysis [57,58,59]. Since polyborazylenes varied in their physical state from liquids to solids, they appeared suitable for various processing and shaping techniques. As an illustration, they were soluble in polar solvents providing an interesting source for shaping in solution such as infiltration of template or preforms as well as dip-coating (Figure 4). When synthesized in the solid state, polyborazylene are well adapted to plastic-forming techniques such as warm-pressing to prepare monolithic pieces (Figure 4).

Table 1 reports some of the characteristics of the polyborazylenes reported by our group [57,58,59]. The characteristics of the polyborazylene are closely related to the temperature of thermolysis. These characteristics impose the selection of the shaping process type. This is well indicated in Table 1. Below, we detail the different shaping processes (Figure 4) which have been applied on the polyborazylene described in Table 1.

Using preceramic polymers for interfaces or protective coatings offers a process with potential for reduced cost compared with conventional chemical vapor deposition techniques. It allows for easy dip-coating of substrates or fibers from dilute solution of a polymer (or pure liquid polymer) which are then pyrolyzed to obtain the desired supported ceramic. As an illustration, liquid polyborazylenes obtained from condensation of borazine at 45 °C (PB45, Table 1) could be dip-coated on metallic (titanium, aluminum and copper) substrates to form protective BN coatings over metallic substrates after pyrolysis by infrared irradiation [57]. Volatilization of a part of the polymer occurred during the pyrolysis leading to a very thin coating with an uncontrollable thickness. The X-ray diffraction confirmed the formation of a poorly crystallized BN which rendered the coating air- and moisture-sensitive.

**Table 1:** Characteristics of the polyborazylenes produced by self-condensation of borazine in an autoclave under argon [57,58,59].

Samples	T_Thermolysis_ (°C)	Physical state	Shaping process type	Empirical formulae ^(1)^	Weight loss (%) ^(2)^
PB45	45	Liquid (extremely volatile)	Solution-based shaping	[B_3.0_N_3.0_H_4.8_]_n_	70
PB50	50	Liquid (volatile)	Solution-based shaping	[B_3.0_N_3.8_H_4.0_]_n_	53.2
PB60	60	Solid	Plastic-forming technique	[B_3.0_N_3.5_H_4.5_]_n_	8.8

^(1)^ Referenced to B_3.0_; Oxygen values < 2 wt%; ^(2)^ Weight loss measured by TGA performed in a nitrogen atmosphere up to 1000 °C (5 °C·min^−1^).

**Figure 5:**
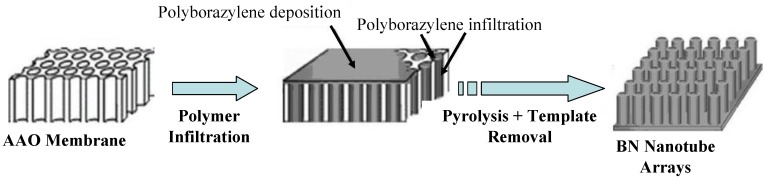
Polymer nanocasting leading to BN nanotube arrays.

Liquid polyborazylene obtained by thermolysis of borazine at 50 °C (PB50, Table 1) [58] are also appropriate for BN coatings but volatilization still occurred during ceramic conversion. Volatilization of oligomers was a key parameter to prepare highly ordered BN nanotube arrays by using template synthesis (Figure 5) [58]. Our strategy is detailed below.

The so-called nanocasting, *i.e.*, hard-template methodology, provides access to design different morphologies of PDCs by (i) replicating the porous structure of a template material through the impregnation of the pore template with a preceramic polymer solution or melt; (ii) performing the subsequent pyrolysis then (iii) removing the mold to form the negative replicas of the template. Nanocasting is mainly applied to prepare porous PDCs [70,71].

In the present case, polyborazylene filled the straight cylindrical pores of a nanoporous anodic aluminum oxide (AAO) membrane (pore size = 200 nm) to form arrays of mono-dispersed and highly ordered nanowires. The pyrolysis at 1200 °C under nitrogen involved gas release and evaporation of low molecular weight species which are responsible of the loss of nanowire integrity. Indeed, the core of the nanowire was decomposed during pyrolysis while a thin film remained and covered the pore walls as a result of the high surface energy of the alumina mould. BN nanotube arrays were generated after membrane dissolution in acid (Figure 5). They displayed a turbostratic structure which crystallized by further heat-treatment above 1200 °C under nitrogen. Nanotubes produced at 1800 °C even showed distinctly ordered wall structures similarly to the tubular morphologies of multiwalled BN nanotubes.

**Figure 6:**
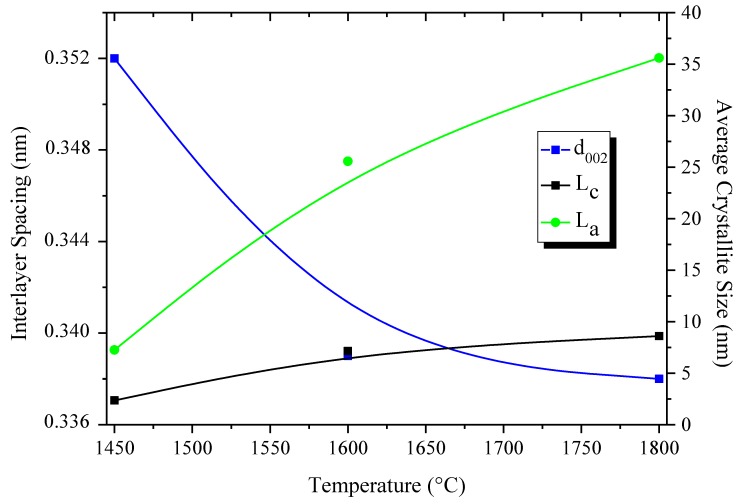
Evolution of the average crystallite sizes and interlayer spacing *vs.* annealing temperature.

As mentioned above, polymer nanocasting is a versatile technique which may be also applied to prepare different morphologies of BN. This has been demonstrated by Sneddon *et al.* [72] through the preparation of nanostructured, nanocrystalline BN microparticles with diatom frustule-derived 3-D morphologies from a dilute solution of solid polyborazylene. These materials as well as those with a porous structure are not the focus of the present review. They have been reviewed elsewhere [73]. Finally, solid polyborazylenes are probably the most appropriate candidates for shaping processes. Indeed, they are well soluble in polar solvents such as THF or glyme. For example, they can be used to prepare coatings and nanowires through diluted solutions of polyborazylene by dip- or spin-coating and nanocasting, respectively. Furthermore, volatilization is prevented during pyrolysis. Therefore, ceramic conversion is relatively well controlled. We used solid polyborazylene synthesized at 60 °C (PB60, Table 1) to prepare highly dense monolithic BN (disc-shaped BN) after pressureless pyrolysis [59]. The polyborazylene exhibited adjusted viscoelastic properties and sufficient plasticity to be warm-pressed under 74 MPa at 60 °C into disk-shaped bodies. Then, the low weight loss of PB60 (8.8 wt% measured at 1000 °C, Table 1) associated with the relatively low variation of the density from polyborazylene to BN allowed us to control the structural integrity of the products after the polymer-to-ceramic conversion at 1450 °C in a nitrogen atmosphere despite an evident volume shrinkage. Disk-shaped turbostratic BN (B_3.0_N_2.95_) with a relative density of 86.3% were obtained after pyrolysis to 1450 °C. Further annealing to 1800 °C induced relative density increase (93%) and crystallization (Figure 6). Figure 6 illustrate the improvement of the crystalline quality and crystallinity *versus* the temperature of elaboration through the evolution of the density, interlayer spacing and average crystallite sizes of the disk-shaped BN. The interlayer spacing d_002_ is calculed from Bragg’s law using the diffraction angle of the (002) peak. The average crystallite sizes L_c_ (in the direction perpendicular to hexagons) and L_a_ (size of BN domains along the sixfold ring plane) are calculated from the Scherrer relation. It should be mentioned that the temperature of elaboration does not only affect these properties. It plays also a key role in the evolution of the thermal conductivity of BN. The thermal conductivity is an interesting property of *h*-BN [74]. This parameter, which is mainly due to phonon, depends very much on the microstructure organization, the porosity and the grain boundary phases. We have followed the variation of the thermal diffusivity as a function of the applied temperature.

**Figure 7:**
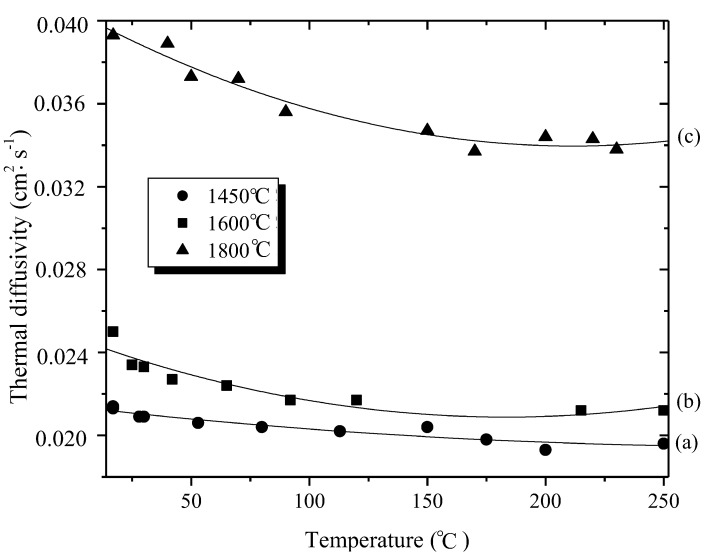
Temperature dependence of the thermal diffusivity of typical disk-shaped samples with warm-pressing pressure under 74 MPa at 60 °C (dwelling time of 0.5 h) and pyrolyzed (a) at 1450 °C; (b) at 1600 °C; and (c) at 1800 °C.

Figure 7 reports the temperature dependence of the thermal diffusivity of the bulk B_3.0_N_2.95_ prepared at 1450 °C (a) and samples obtained at 1600 (b) and 1800 °C (c). We measured the thermal diffusivity in the temperature range 15–250 °C and we performed tests in the direction parallel to the warm-pressing direction. The variation of the thermal diffusivity of each sample with the test temperature is similar. It decreases with increasing test temperature which is related to the increased phonon scattering due to the higher degree of anharmonicity [75]. In addition, it was observed that the thermal diffusivity increases from samples prepared at 1450 °C to those prepared at 1800 °C, thereby when crystalline ordering increases. Samples prepared at 1450 °C which exhibit a turbostratic structure show the lowest thermal diffusivity in the temperature range 15–250 °C, whereas disk-shaped boron nitride prepared at 1800 °C displays the highest thermal diffusivity over the entire temperature range. Since the phase compositions of each sample are close, we attributed the difference in thermal diffusivity to microstructural variations. The thermal conductivity K (in W·m^−1^·°C^−1^) at RT was calculated based on the heat capacity of hexagonal-boron nitride (791.6 J·kg^−1^·°C^−1^ at 300 K) [76,77], the thermal diffusivity α measured at RT by the laser-flash technique (in cm^2^·s^−1^) and reported in Figure 7 and the bulk density ρ (g·cm^−3^) of the material according to the equation K = Cp × α × ρ [75]. Increasing the temperature of preparation increased the thermal conductivity from 3.26 W·m^−1^·°C^−1^ for the samples prepared at 1450 °C to 6.56 W·m^−1^·°C^−1^ for the samples prepared at 1800 °C.

**Figure 8:**
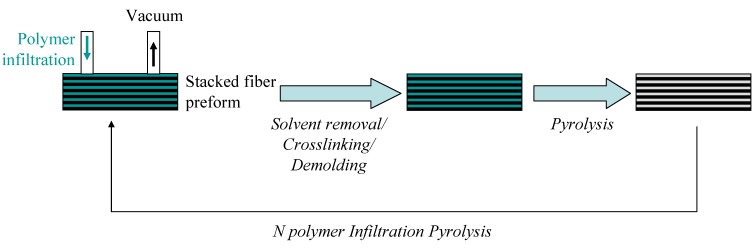
Polymer infiltration pyrolysis (PIP) to prepare fiber-reinforced ceramic matrix composites.

The main problem of monolith is their brittleness. The introduction of long carbon or ceramic fibers in these monoliths to form fiber-reinforced ceramic matrix composites has drastically increased the crack resistance, elongation and thermal shock resistance, and resulted in several new applications. Economy *et al.* [67] reported in 1993 the preparation of C/BN composites through one or two step impregnation of a liquid polyborazylene into a mold containing carbon fiber preforms. After impregnation within the porous structure of the carbon fiber preforms, the C/polyborazylene composite was heated up to 60–70 °C before pressed at 400 °C. Finally, samples were pyrolyzed in the temperature range 1200–1500 °C to generate C/BN composites. Then, the same group published several papers in which they focused on the characterization of C/BN composites, *i.e.*, thermal stability and mechanical properties (Friction and wear properties) [78,79,80,81]. In our group, carbon fibers-reinforced BN matrix composites (C/BN composites) were manufactured after 10 polymer impregnation pyrolysis (PIP) cycles with PB60 (Table 1, first PIP cycles) and PB50 (Table 1, last PIP cycles) and dissolved in THF [82]. PIP is a method of fabrication of ceramic matrix composite parts from dilute solutions of preceramic polymers (Figure 8).

During PIP, the fiber preform is immersed into the polymer and the latter infiltrates the porosity of the former. The infiltration process is driven by the capillary forces therefore it is commonly conducted at normal pressure. However, it may also be vacuum-assisted as we aimed to prepare carbon fiber-reinforced BN matrix composites (C/BN). The green composite is then pyrolyzed in inert or reactive environment at elevated temperatures. Owing to shrinkage of the polymer during the decomposition to ceramic, multiple PIP cycles are required to produce parts of adequate density. To prepare C/BN composites, the first PIP cycles were performed with PB60 (Table 1) because of its solubility, adapted viscosity when diluted in THF to infiltrate fiber preforms and high ceramic yield. However, the viscosity of the diluted solution of PB60 (Table 1) was too high after five PIP cycles to further infiltrate the fiber preforms. Therefore, we used diluted solutions of PB50 (Table 1) which displayed a lower viscosity for the next five cycles. After each infiltration, the curing and pyrolysis processes are achieved at 85 °C and 1450 °C, respectively. C/BN composites exhibited a relative density of 94.7% and an open porosity as low as 5.1 vol%.

**Figure 9:**
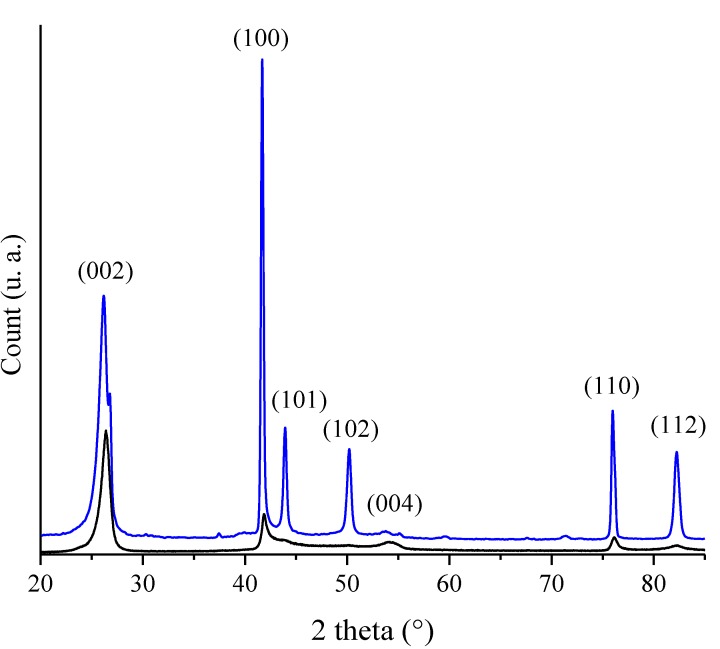
XRD patterns of monolithic BN prepared following the strategies 1 (warm-pressing of PB60 → pyrolysis, black curve) and 2 (spray-pyrolysis of borazine → hot-pressing, blue curve).

We have seen the high potential of polyborazylene to prepare BN in various shapes using solution-based shaping process (infiltration, dip-coating) or plastic-forming techniques (warm-pressing) followed by pyrolysis. Based on the results reviewed previously, we can suggest that polyborazylene (and therefore borazine) are among the most appropriate molecular candidates to prepare BN at relatively low temperature in an inert atmosphere. It should be mentioned that we may also apply the strategy 2 (Figure 1b) which consists of directly converting borazine into BN nanopowders (without polyborazylene intermediates) before a further shaping/sintering process to produce nearly dense materials [83]. This combined approach is expected to offer many advantages with a major one: we can prepare monoliths which replicate the cylindrical shape of the fabrication die to generate ceramics with relatively well controlled dimensions (=without any volume shrinkage). Typical consolidation methods such as hot pressing (HP), hot isostatic pressing (HIP) and Spark Plasma Sintering (SPS) are mostly used for the densification of non-oxide ceramics derived from molecular precursors and preceramic polymers [45,46,47,48,49]. Following this strategy, we combined the spray-pyrolysis process of borazine to produce BN nanopowders and an additive-free sintering at 1840 °C under nitrogen of the latter leading to bulk BN with a very high relative density (96.3%), an homogeneous microstructure, an average hardness throughout the cross-section of 8 Hv (78 MPa) [83]. In comparison to the strategy 1 (Figure 1a) which combines warm-pressing of polyborazylene (PB60, Table 1) and pyrolysis to 1800 °C as previously described, BN samples without volume shrinkage and highly crystallized and oriented are generated as shown through the XRD pattern of the two samples in Figure 9.

Finally, borazine and the derived polyborazylenes offer the possibility to imagine a relatively large panel of shapes by considering all potential shaping processes in gas, liquid and solid (=plastic) states. However, according to the presence of reactive BH and NH units, the melt-processability of polyborazylene is not possible because polyborazylene crosslinks before melting. As a consequence, the production of fibers which is best achieved by melt-spinning of preceramic polymers is not possible.

**Figure 10:**
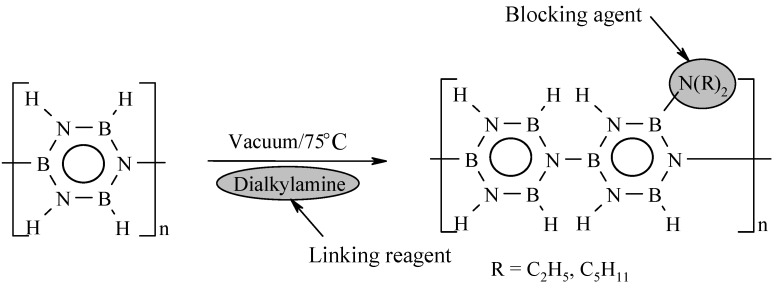
Amine modification of polyborazylenes.

Since the initial cross-linking reactions, *i.e.*, dehydrocoupling, during synthesis and conversion of polyborazylene do not allow considering applications requiring a melt-processable polymer, studies have been focused on the control of these reactions, thus the degree of cross-linking, through chemical modification of polyborazylene. For this purpose, Sneddon *et al.* [65,84] demonstrated that the preparation of dialkylamine-modified polyborazylenes was an interesting strategy for obtaining melt-processable polyborazylenes. Their approach consisted in grafting dialkylamino substituents on the borazine ring, thereby blocking some reactive BH sites in order to shift the cross-linking reactions and decrease the molecular weight of polyborazylene. In typical conditions, polyborazylene and dialkylamine were dissolved in glyme and heated at 75 °C under vacuum (Figure 10).

The processability of the resulting modified polyborazylene was clearly improved using pentyl as alkyl group. The dipentylamino substituents blocked the cross-linking progress at low temperature during thermolysis. As a consequence, dipentylamine-modified polyborazylene became fluid without enhanced cross-linking or decomposition upon heating from 75 to 95 °C. Consistently, the glass transition temperature *T*_g_ was identified below the cross-linking temperature, in contrast to polyborazylene.

It was thus proposed that dipentylamino groups acted as plasticizers to render the polyborazylene more tractable in the temperature range 75–95 °C, and even spinnable in the molten state. Thus as-spun fibers could be cured in air before pyrolysis to 1000 °C in an ammonia atmosphere to obtain carbon-free BN fibers 30 µm in diameter in a ceramic yield which is significantly reduced (~64%) in comparison to that of polyborazylene (85%–93%). This is caused by a lower degree of cross-linking and the presence of a high-weight leaving group, *i.e.*, dipentylamine, instead of hydrogen in the polymer network. These observations highlighted the strategy that must be conducted for processing BN in particular shapes *via* melt-processing. It is focused on the introduction of specific structural motifs in the initial molecular structure and/or in the polymer backbone which (*i*) possess a certain thermal stability at low temperature to slow down the progress of cross-linking reactions and shift them above the polymer melting point, and (*ii*) offer flexibility and plasticity to the polymer backbone.

The introduction of alkylamino groups at molecular scale in BN precursors appeared to be the best solution up to now to develop the melt-processing of borazine-based precursors. Such precursors are called B-tri(methylamino)borazines. These precursors with their polymeric derivatives are described in the following section.

## 3. B-trichloro-/B-tri(methylamino)-Borazine and Poly[B-tri(methylamino)borazine-Derived BN

The processability of preceramic polymers in their molten state is of scientific and technological importance in the field of PDCs. Indeed, melt-processing is the main process to produce PDC fibers. The process, originally derived from the method described by Yajima involves the melt-spinning of preceramic polymers followed by a curing step and subsequent pyrolysis of the polymer fiber providing the ceramic fiber [85,86,87]. The intermediate curing step results in an increased cross-linking of the polymer and hence in the formation of an infusible polymer fiber necessary for the shape retaining thermal conversion into the ceramic material. Many examples of polymer-derived ceramic fibers have been reported through reviews or specific articles [25,26,28,29,36,37,85,86,87,88,89,90,91,92,93,94,95,96,97,98,99,100,101,102,103,104,105,106,107,108,109,110,111,112,113], in particular on BN fibers by our group [101,102,103,104,105,106,107,108,109,110,111,112,113].

**Figure 11:**
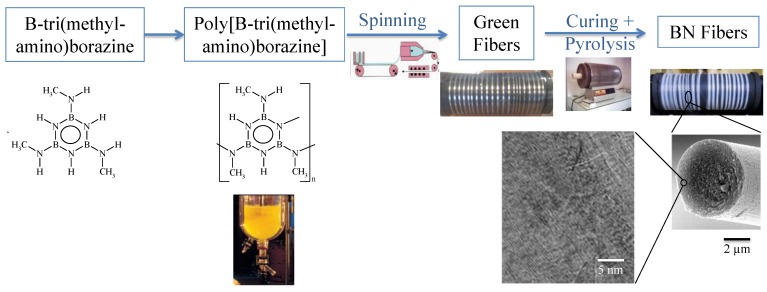
Overall process for preparing BN fibers: polymer melt-spinning, curing and pyrolysis.

Poly[B-(amino)borazine] (or poly[borazinylamine]) and poly[B-(alkylamino)borazine] have demonstrated to be well suited for filling the requirements as BN fiber precursors. A lot of works devoted to polymer-derived BN fibers have been reported on these polymers [101,102,103,104,105,106,107,108,109,110,111,112,113,114,115,116,117]. Among the different polymer systems which have been described, poly[B-tri(methylamino)borazine] derived from B-tri(methylamino)borazine in which methylamino units are linked to boron atoms are the most appropriate compounds in terms of spinnability and ceramic yield to produce BN fibers with relatively high performance (Figure 11) by melt-spinning, curing and pyrolysis processes [101,102,103,104,105,106,107,108,109,110,111,112,113,117].

**Figure 12:**
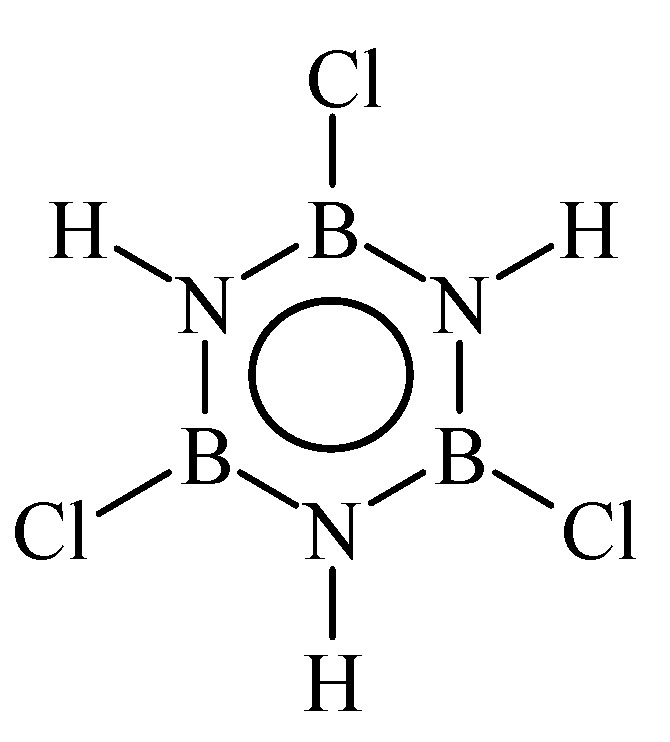
Structure of B-trichloroborazine.

Unlike synthesis of polyborazylene, the preparation of poly[B-tri(methylamino)borazine] is much more complex. It involves two steps (aminolysis with methylamine and thermolysis) starting from B-trichloroborazine (Figure 12).

B-Trichloroborazine (ClBNH)_3_ [118] is a white solid compound more stable than borazine and has been recognized as a desirable precursor for the preparation of BN fibers. However, B-trichloroborazine is corrosive when exposed to humid air because it can hydrolyze and release HCl. As borazine, it is therefore best stored at low temperatures and handled under an inert atmosphere. The two-step conversion of B-trichloroborazine into poly[B-tri(methylamino)borazine] particularly requires a precise control of the second step to develop the melt-spinnability of poly[B-tri(methylamino)borazine]. The latter are in general obtained in the temperature range 140–200 °C by thermolysis of B-tri(methylamino)borazine under inert atmosphere. It appeared that the most appropriate candidates to be melt-spun are poly[B-tri(methylamino)borazine] synthesized by thermolysis of B-tri(methylamino)borazine in the temperature range 160–185 °C [109,110,111,112]. These melt-spinnable polymers display a chemical formula of [B_3.0_N_4.4 ± 0.1_C_2.0 ± 0.1_H_9.3 ± 0.2_]*_n_* (*n* ~ 7.5) and glass transition temperatures in the range 64–83 °C. However, it was demonstrated that the preparation of BN fibers does not only depend on the melt-spinning process. It is also affected by the weight loss which is imposed by the polymer during the subsequent curing and pyrolysis processes around the spool [109]. Indeed, the quality of the BN fibers depends on the control of the four-step weight loss which occurs under ammonia in the temperature range 25–1000 °C [101,113]. Pyrolysis under ammonia involves the elimination of the organic moieties typical of poly[B-tri(methylamino)borazine] (amine, ammonia, *etc.*) with as a consequence significant gas release and shrinkage. Shrinkage involved the loss of structural integrity of the fibers prepared from poly[B-tri(methylamino)borazine] synthesized below 170 °C. In contrast, poly[B-tri(methylamino)borazine] synthesized at 170 and 180 °C combined appropriate rheological properties and sufficient ceramic yield to lead to BN fibers with tensile strength above 1.4 GPa after pyrolysis at 1800 °C.

**Figure 13:**
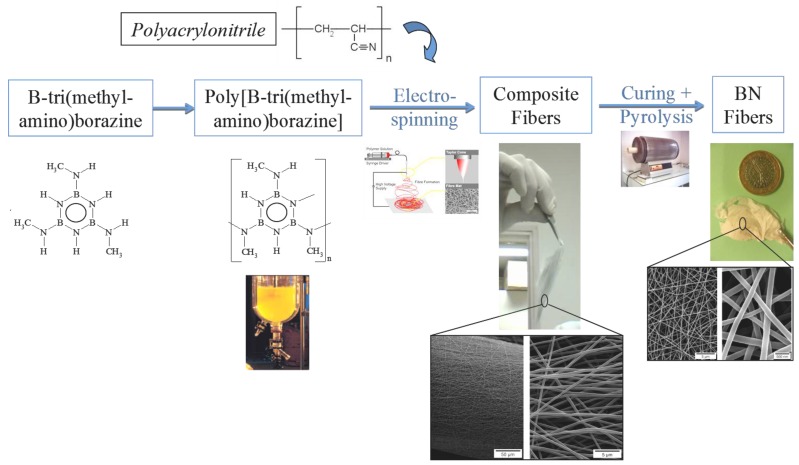
Overall process for preparing BN fibers by electrospinning, curing and pyrolysis.

Interestingly, poly[B-tri(methylamino)borazine] are also soluble compounds in toluene or dimethylformamide (DMF) and can be mixed with high molecular weight and linear polymers such as polyacrilonitrile (PAN) to prepare BN nanofibers by electrospinning then curing and pyrolysis according to the processing scheme depicted in Figure 13 [119].

Electrospinning is a versatile method to produce functional nm to µm fibers of polymers, ceramics or composite materials and is used in the processing of fibers for various applications in fields such as energy, healthcare and environmental engineering. For the production of ceramic fibers, electrospinning can be achieved in the molten state or in solution. However, in the majority of cases, electrospinning conventionally consists of the spinning of a mixed solution of an inorganic precursor and an organic polymer. The latter serves as the structural matrix for optimized viscoelastic behaviour of the inorganic polymer to avoid the formation of droplets (electrospraying).

By appropriately controlling the ratio between the inorganic (poly[B-tri(methylamino)borazine]) and organic (PAN) parts as well as between the hybrid polymer and the solvent, a felt composed of entangled composites fibers with relatively low diameter can be formed by electrospinning. It should be mentioned that electrospinning parameters are also important to succeed in the elaboration of a felt. After the spinning process, the polymer fibers composing the felt is cured in an infusible form then pyrolyzed according to the process developed for BN fibers obtained by melt-spinning. Curing and pyrolysis are achieved under ammonia to 1000 °C. Ammonia allowed (i) curing fibers by transamination reactions substituting methylamino groups with ammonia, which increased cross-linking within the polymeric network and (ii) removing PAN from the hybrid polymer to prepare carbon-free BN fibers. A relatively large shrinkage of the felt occurred but structural integrity of the fibers was in general well controlled. This is probably due to the intrinsic short diffusion paths for the generated gases leading to very limited internal pressure build-up [120]. In a second step, a pyrolysis at 1800 °C under nitrogen allows generating BN submicro- and nano-fibers in forms of a felt or a membrane. According to the solubility of solid polyborazylene (PB60, Table 1), it could be electrospun then pyrolyzed to produce membranes most probably with a limited shrinkage. Such works are under investigation. We expect application as catalyst support or for water treatment.

As previously seen, the two main disadvantages of poly[B-tri(methylamino)borazine] are (i) their weight loss occurring during the pyrolysis under ammonia to 1000 °C (generally in the range 45%–55%) and (ii) the necessity to increase the final temperature to 1800 °C to obtain BN with a sufficient degree of crystallinity. The ceramic yield of polyborazylene can be significantly higher and there is no need for reactive gas during the pyrolysis or high temperatures to induce a sufficient degree of crystallization. Based on such a discussion, polyborazylene appears to be the ideal BN precursor for shaping and shape retention during pyrolysis. However, poly[B-tri(methylamino)borazine] is both soluble and fusible which is not the case of polyborazylene. This highlights that combining all the properties required preparing BN and more generally PDCs in various shapes, *i.e.*, solubility, fusibility, high ceramic yield, in only one preceramic polymer is extremely ambitious and generally not possible. Compromises are needed and molecular/inorganic chemistry is clearly the solution to do these compromises.

## 4. Conclusions

We have seen that hexagonal boron nitride (BN), a III-V compound which is the focus of important research since its discovery in the early 19th century, displays a combination of unique properties that depends on the synthesis of BN. This review stated the recent developments in the preparation of BN through the synthesis, shaping and ceramic conversion of borazine-based precursors.

The effect of the chemistry of the molecular precursors, *i.e.*, borazine and trichloroborazine, and their polymeric derivatives *i.e.*, polyborazylene and poly[tri(methylamino)borazine], on the shaping and pyrolysis processes has been reviewed. The possibility to tailor the structure of these precursors through the synthesis parameters offers the possibility to apply a solution-based shaping process such as infiltration, dip-coating and electrospinning as well as plastic-forming techniques such as warm-pressing and melt-spinning to prepare a large variety of BN shapes which inherently extend the application of BN. The different examples presented in this review show that combining all the properties required to prepare BN in various shapes, *i.e.*, solubility, fusibility, high ceramic yield, in only one polymer is generally not possible. Compromises are needed and molecular/inorganic chemistry is clearly the solution to make these compromises and prepare BN in a large variety of shapes. The application of these polymer-derived BN products is poorly reported. This is the main limit of this approach. However, according to the different applications which are reported for BN produced through more conventional synthesis approaches, it is anticipated that polymer-derived BN could propose specific functionalities as well as improved and/or extended properties to be applied in a large variety of application field. These opportunities are now being addressed.
